# Effect of Stirring Pin Rotation Speed on Microstructure and Mechanical Properties of 2A14-T4 Alloy T-Joints Produced by Stationary Shoulder Friction Stir Welding

**DOI:** 10.3390/ma14081938

**Published:** 2021-04-13

**Authors:** Haifeng Yang, Hongyun Zhao, Xinxin Xu, Li Zhou, Huihui Zhao, Huijie Liu

**Affiliations:** 1 State Key Laboratory of Advanced Welding and Joining, Harbin Institute of Technology, Harbin 150001, China; hf_yang@hit.edu.cn (H.Y.); zhaohy6691@hit.edu.cn (H.Z.); liuhj@hit.edu.cn (H.L.); 2 Shandong Provincial Key Laboratory of Special Welding Technology, Harbin Institute of Technology, Weihai 264209, China; yysong@hit.edu.cn; 3 Shanghai Aerospace Equipments Manufacturer Co., Ltd., Shanghai 200245, China; 18b909129@stu.hit.edu.cn

**Keywords:** 2A14-T4 aluminum alloy, SSFSW, T-joint, microstructure, mechanical properties

## Abstract

In this study, 2A14-T4 Al-alloy T-joints were prepared via stationary shoulder friction stir welding (SSFSW) technology where the stirring pin’s rotation speed was set as different values. In combination with the numerical simulation results, the macro-forming, microstructure, and mechanical properties of the joints under different welding conditions were analyzed. The results show that the thermal cycle curves in the SSFSW process are featured by a steep climb and slow decreasing variation trends. As the stirring pin’s rotation speed increased, the grooves on the weld surface became more obvious. The base and rib plates exhibit W- or N-shaped hardness distribution patterns. The hardness of the weld nugget zone (WNZ) was high but was lower than that of the base material. The second weld’s annealing effect contributed to the precipitation and coarsening of the precipitated phase in the first weld nugget zone (WNZ1). The hardness of the heat affect zone (HAZ) in the vicinity of the thermo-mechanically affected zone (TMAZ) dropped to the minimum. As the stirring pin’s rotation speed increased, the tensile strengths of the base and rib plates first increased and then dropped. The base and rib plates exhibited ductile and brittle/ductile fracture patterns, respectively.

## 1. Introduction

Aluminum alloy T-joints, as integral parts of load-bearing structures, can bear both unidirectional and combined stresses, thereby effectively improving the rib plates’ stability without affecting the component’s overall quality. Despite extensive applications in aeronautics, astronautics, and railway vehicles [1,2], aluminum alloy T-joints produced by riveting, extrusion forming, traditional fusion welding, or bonding still have numerous deficiencies, including limited mold shape, residual stress concentration, and burning loss of alloying elements [3,4,5,6].

Friction stir welding (FSW) is a novel solid-state bonding technique developed by The Welding Institute (TWI, Cambridge, UK) [7,8]. It possesses many advantages, such as low thermal input, slight residual deformation, high material utilization, and favorable automation degree during the butt welding of aluminum alloy plates [9,10]. T-joints can generally be prepared via FSW by passing through the rib plate to the base plate [11,12]. However, the joint binding rate is low, and a right angle tends to easily form at the connection between the base and rib plates, thereby leading to serious stress concentration [13]. Yang et al. [14,15,16], classified defects in Al-alloy FSW T-joints as tunnel-type, weak-binding, and Z-line defects. In 2005, TWI proposed stationary shoulder friction welding (SSFSW) solid-phase joining technique [17]. SSFSW has a unique advantage in the welding of T-joint welded joints, i.e., the welding can start from the inside of the joint only by processing the shaft shoulder into a special rectangular shape.

Penalva et al. [18] designed a soldering set for SSFSW of T-joint Al-alloy joint and successfully obtained SSFSW T-shape joints. However, on account of the stirring pin’s suction effect, the grooves and tunnel-typed defects inevitably existed, accompanied by the fracture of the stirring pin. Martin et al. [19] prepared T-joint Al-alloy joints via SSFSW, which showed smooth surface forming, no thinning of the welding zone, narrow heat-affected zone (HAZ), and small post-welding deformation. Gascoyne et al. [20] analyzed the microstructures of the SSFSW T-joints. They found that, the appearance of blades is connected with the design structure of the tool and the thread of the stirring pin, which also certainly depends on the welding parameters and the properties of the base materials. Buffa et al. [21] elaborated on temperature field modeling using the design environment for forming (DEFORM) software and reported that the obtained temperature field was close to that of the low-temperature shaft shoulder outside the joint. Ji et al. [22] performed the T-joint 6061-T6 Al-alloy joints’ welding via SSFSW and found that the T-joint weld joint surface was well-formed without surface scratches or furrows at a rotation speed of the stirring pin of 1600–2200 rpm and a welding speed of 40–80 mm/min. According to Li et al.’s [23] research results, the T-joint weld joints via SSFSW were improved in fatigue compared with the T-joint using the traditional welding method. Hao et al. [24] designed an SSFSW welding tool for T-joints, including the stirring pin with different structures. Accordingly, the stirring pin could well stir plastic materials; however, some details, such as structural sizes, were omitted.

Based on the public reports regarding SSFSW T-joints, the welding tool’s design details, the transition characteristics of the structural properties, and the joint’s fracture behavior need further investigation. This study performed SSFSW T-joint welding tests on 2A14 Al-alloy. It examined the forming quality on the weld surface, the evolution of microstructures, and the related mechanical properties at different rotation speeds in-depth, which can provide insight into experimental and theoretical foundations for achieving high-quality bonding of Al-alloy T-joints via the SSFSW.

## 2. Materials and Methods

2A14-T4 Al-alloy, as a kind of Al-Mg-Si-Cu high-strength Al-alloy, was used in the present experiments. The base plate, with a size of 300 mm × 200 mm × 8.5 mm, and the rib plate, with a size of 300 mm × 100 mm × 8.5 mm were used. Their chemical components and mechanical properties are shown in Table 1.

The temperature field variations at feature point in the base and rib plates (namely, located at 15 mm from the welds’ center) during the welding process were measured by a WZ-CP16 multi-channel temperature meter (Huipu, Hangzhou, China) and GG-K-30 thermocouples (Huipu, Hangzhou, China). The temperature field distribution patterns at different regions in the SSFSW-welded T-joint under different technologic conditions were simulated using the abaqus commercial software package (version: 6.14, 2014, ABAQUS Inc., Palo Alto, CA, USA). Considering the complex thermodynamic variation during the welding process, the plastic deformation heat of materials and the latent heat of phase change during the structural transformation were ignored. In contrast, only the friction heat was taken into account. The simulation’s geometrical model mainly consisted of two parts: the materials to be welded and the shaft shoulder. The entire welding process can be simplified as follows to enhance the computational speed and satisfy model convergence requirements. By neglecting the upper part of the shaft shoulder’s characteristics, only the lower part of the shaft shoulder and the part adhesive to the materials to be welded are considered in calculations. The T-joint dimensions were as follows: the base plate was 300 mm in length, 200 mm in width, and 8.5 mm in thickness. The rib plate was 300 mm in length, 100 mm in width, and 8.5 mm in thickness. The welding was performed along the Y-axis. The T-joint and shaft shoulder were endowed with 2A14 alloy and H13 steel cross-sectional material properties.

**Figure 1:**
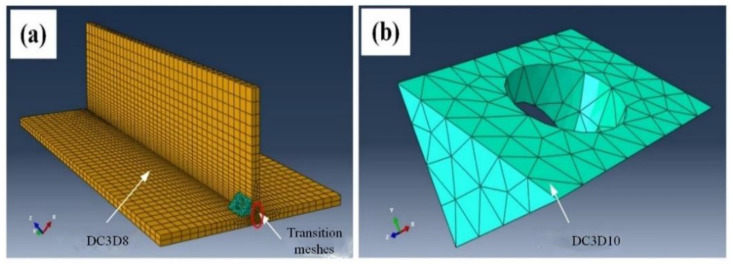
Mesh generation results of the geometrical model: (**a**) meshed material to be welded; (**b**) meshed stationary shoulder.

To optimize computations, several different types of meshes were adopted. The material to be welded was meshed using eight-node linear heat-transfer hexahedral elements (DC3D8). To ensure high computing precision and simultaneously enhance the operating efficiency, inhomogeneous meshes were also applied. In particular, fine structural meshes were generated near the weld zone, sparse meshes were generated far away from the weld zone, and transitional meshes with the size from 1 mm × 1 mm to 5 mm × 5 mm were generated in the middle. In general, mesh elements in total were generated in the material to be welded. Besides, uniform meshes were generated in the shaft shoulder using 10-node secondary heat-transfer tetrahedron elements (DC2D10), in view that no heat was produced during the welding process. One thousand ninety-eight meshes in total were generated in the shoulder part. Figure 1 shows the mesh generation results.

In general, the value of the physical parameters of the material will change with the change of temperature. The SSFSW welding process is a process of local heating to the peak temperature and then natural cooling. As the moving heat source moves along the welding direction, the temperature in different areas of the T-joint changes at any time. The specific values of physical properties of 2A14 aluminum alloy changing with temperature are shown in Table 2 and Table 3. In the welding process of SSFSW T-joint, the material is affected by temperature and large plastic deformation produced. The elastoplastic model is adopted for the establishment of the finite element method (FEM) modeling. The Johnson–Cook constitutive equation parameters of 2A14-T4 Al-alloy in the SSFSW welding process of large plastic deformation at high temperature and high strain are shown in Table 4.

In order to realize the movement of the SSFSW welding heat source and the corresponding constraints of the welding material. It is necessary to apply the corresponding load and the predefined field. The body heat flux of magnitude 1 was set as the load condition for heat conduction in all the analysis steps. The predefined field sets the initial temperature of all components to 25 °C. The air convection heat transfer coefficient was set as 100 W/(m^2^·°C), and plate convective heat transfer coefficient was set as 1000 W/(m^2^·°C) [32,33]. The heat dissipation coefficient between the stationary shoulder and the material to be welded was set as 75 W/(m^2^·°C).

The SSFSW temperature field analysis belonged to the category of nonlinear transient thermal analysis. The thermodynamic coupling method was used to carry out the numerical simulation, and the stirring pin is equivalent to a moving heat source to establish an effective model. The moving heat source was loaded in the shaft shoulder’s hole center at the connection center between the base and rib plates. The heat source model can be described via Equations (1) and (2) [34,35]:

(1) $$Q=\frac{2\pi \omega \tau_{contact}}{3\;sin\beta }\left(R_{p1\;-\;}^{3}R_{p2}^{3}\right)$$

where *Q* is heat production from the stirring pin; *ω* is the rotation speed of the stirring pin (rad/s); *τ_contact_* is shear stress on the contact surface ($\tau_{contact}\;=\;\mu F/\left(\pi R_{p1}^{2}\right)$); *F* is the axial pressure on the stirring pin; *R_p_*_1_ and *R_p_*_2_ are the root and end radius of the tapered stirring pin, respectively; *β* is taper angle of the stirring pin.

The body heat source with uniformly distributed heat flux was set at the stirring pin [34,35,36]. The heat flux of the body source, q, can be calculated as:

(2) $$Q=\frac{Q}{V}$$

where *V* is the insertion volume of the tapered stirring pin.

**Figure 2:**
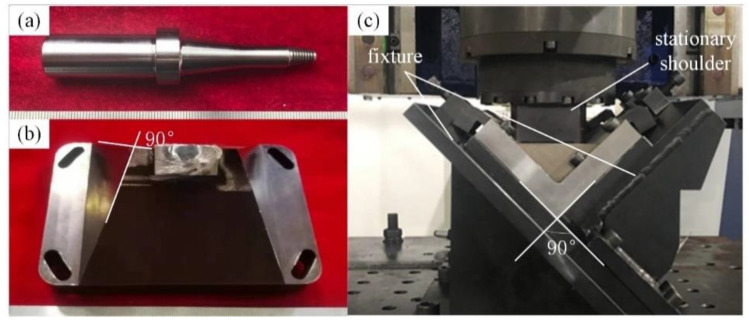
Physical drawing of welding platform: (**a**) SSFSW tool and (**b**) stationary shoulder and (**c**) the welding fixture employed for producing T-joint welds.

The YXR33 stirring pin (Youzhi, Ningbo, China) used in this study was featured by ultra-high strength, ductility, and excellent corrosion resistance. The tapered stirring pin with right-handed screw thread was used to improve the material’s plasticity characteristics, whose total length, root diameter, and front diameter were 92, 10.6, and 7.4 mm, respectively, as shown in Figure 2a. As shown in Figure 2b, the H13 steel was processed on the top of the stationary shoulder at 90°. Before the welding, the base plate and the rib plate were fixed in the 90° fixture. Figure 2c shows the overall assembly of the SFSW process, in which the spacing between the stirring pin and the stationary shoulder was 0.3 mm. After the first weld bead was finished, the welded plate was taken out, overturned, and re-inserted into the fixture. Then, the welded plate was naturally cooled for an hour for the next welding.

The gantry-type friction stir-welding equipment was used in the present experiment. The displacement-controlled welding equipment is easy and simple to operate. During the production of the welds, the pin tool plunge depth was 4.59 mm. Table 5 lists the welding parameters of the adopted welding process.

**Figure 3:**
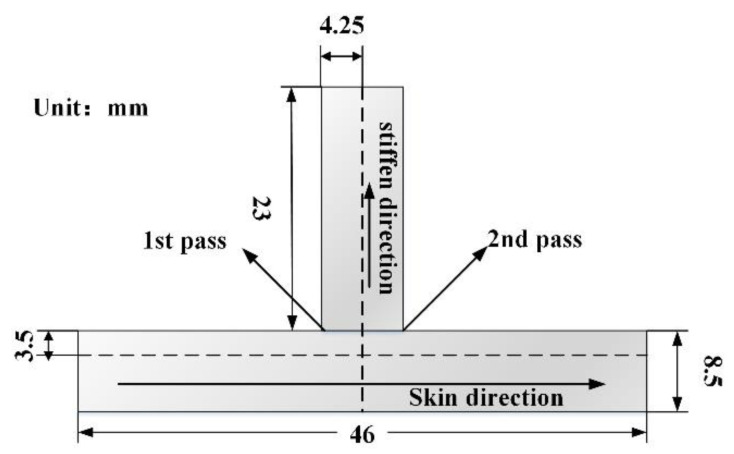
Illustration of the hardness test of SSFSW T-joint.

**Figure 4:**
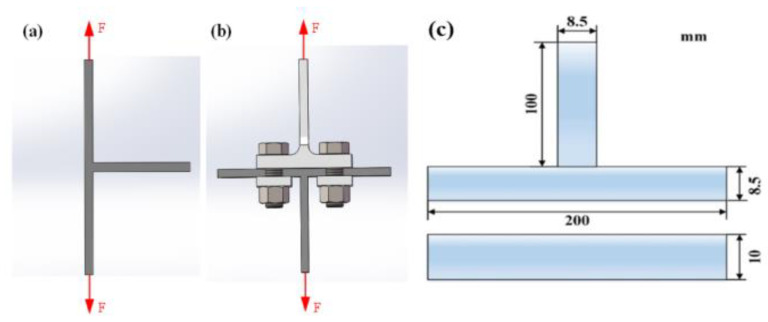
Tensile loading diagrams of SSFSW T-joint: (**a**) along the stringer direction and (**b**) along the skin direction, and (**c**) specimen dimensions.

The metallographic specimen was cut from the welded joint by a DK7745 wire cut electric discharge machine (Ruite, Taizhou, China), then the specimen was eroded by the standard Keller’s reagent for 25–30 s. The microstructures of different regions on the T-joint cross-section were analyzed and observed using an Olympus-DSX510 optical microscope (OLYMPUS, Tokyo, Japan). The distribution of the precipitated phase and the morphology of the tensile fracture of the SSFSW T-joint were observed and analyzed using the Zeiss-MERLIN Compact field-emission scanning electron microscope (SEM) and the energy-dispersive X-ray (EDX) analyser (Zeiss, Heidenheim, Germany). The cross-section of the metallographic specimen after corrosion was tested by the ARTCAN-300SSI-C hardness meter (Hengyu, Suzhou, China). The feature data points along two directions were plotted. Two measuring points (i) 3.5 mm above the floor and (ii) at the rib plate’s centerline were selected. The spacing between the adjacent measuring points was 0.5 mm. As shown in Figure 3, the test load and the pressure-holding time were set as 0.98 N and 15 s, respectively. Figure 4 shows the tensile specimen dimensions. Specimens were tested along with axial directions of the base and rib plates via an INSTRON 5967 electronic universal material tester at a 5 mm/min loading rate (SUNS, Shenzhen, China).

## 3. Results and Discussion

### 3.1. Distribution Characteristics of the Temperature Field in SSFSW T-Joints

**Figure 5:**
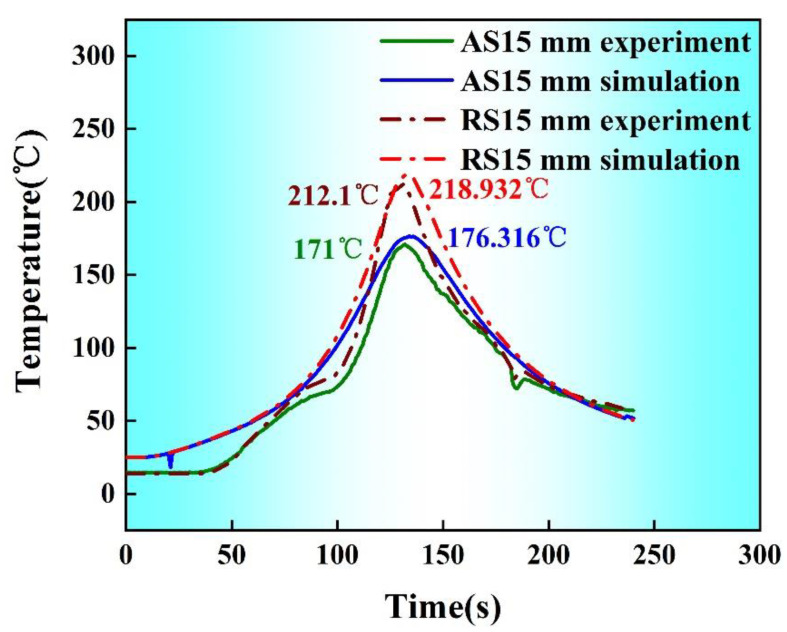
Comparison between the simulated thermal cycle curves and the measured results under typical welding parameters.

Figure 5 shows the comparison between the simulated thermal cycle curves and the measured results of the T-joint welded joint via SSFSW at a rotation speed of 2000 rpm and a welding speed of 50 mm/min as the first welds of the T-joint weld have a nearly identical temperature distribution pattern with the second welds. Therefore, only the temperature distribution field in the first welds was considered. The thermal cycle curves of the feature point featured a steep climb and slow decreasing variation trends. At the pre-heating phase, the temperature slowly rose from the room temperature; when the stirring pin moved closest to the feature point, the temperature at the feature point increased drastically; the welding entered into the stable phase at approximately 135 s, and meanwhile, both the front side and the rear side reached the peak temperature. At the stable welding phase, the material’s peak temperature to be welded was almost unchanged, and the temperature began to drop slowly as the stirring pin moved far away from the feature point.

At the beginning of the welding process, the heat produced during high-speed friction between the stirring pin and the material to be welded can be rapidly transferred on the surface of the material to be welded. At the stable welding phase, the heat produced by the stirring pin was constantly transferred to the surface of the material to be welded. Afterward, under the stirring action, the material to be welded was gradually filled from the front side to the rear side, and the pressure dropped gradually with time; accordingly, the heat generation by the friction of the stirring pin on the material to be welded gradually dropped, and simultaneously, the heat source began to move away from the feature point. Therefore, the temperature dropped slowly after 135 s. It can also be observed that the simulated peak temperatures on both the advancing side (AS) and the retreating side (RS) exceeded the measured data by approximately 5 °C, suggesting that the simulation results fit well with the measured data in the heating phase, at the peak temperature moment and at the end of the welding phase in terms of temperature variation rate and temperature value. Conclusively, the established model can well reflect the temperature field’s change during the SSFSW process of the T-joint.

**Figure 6:**
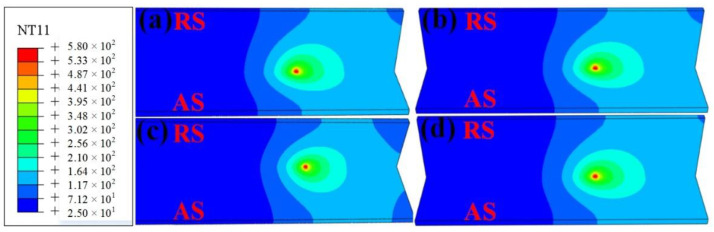
Contours of the temperature fields at different phases in SSFSW process at various rotation speeds: (**a**) 1500 rpm; (**b**) 1750 rpm; (**c**) 2000 rpm; (**d**) 2250 rpm.

Figure 6 shows the surface temperature fields’ distribution patterns at different rotation speeds of the stirring pin, while the welding speed was fixed at 50 mm/min. It can be observed that the temperature field extended towards the two sides in the elliptical pattern around the welds at various rotation speeds. The high-temperature region of the stirring pin increased with rotation speed. The temperature at the edge of the material to be welded also rose steadily. The high-temperature region was mainly distributed at the intersection between the base plate and the stirring region. Meanwhile, the highest temperatures of 427.1, 491.6, 545.4, and 561.1 °C, respectively, increased gradually with rotation speed. The increase in the rotation speed enhanced the thermal input, and therefore, more heat could be diffused at the edge of the material to be welded. By contrast, the heat dissipation condition at the back of the stirring pin was poorer than that in front of it, and the temperature was more easily accumulated in the region.

### 3.2. Rotation Speed Effect on the Surface of SSFSW T-Joints

**Figure 7:**
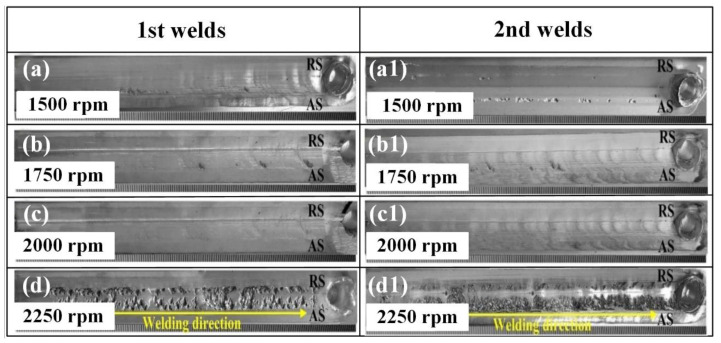
Surface morphologies of the 1st and 2nd welds at different rotation speeds: (**a**)–(**a1**) 1500 rpm; (**b**)–(**b1**) 1750 rpm; (**c**)–(**c1**) 2000 rpm; (**d**)–(**d1**) 2250 rpm.

Figure 7 shows the forming conditions at the first (1st) and second (2nd) welds when the welding speed was at 50 mm/min, while the stirring pin’s rotation speed changed. At low rotation speeds, a small number of grooves appeared on the surface of the first and second welds. As the rotation speed increased to 2000 rpm, the first and second welds had smooth surfaces, and no surface defects appeared. However, as the rotation speed of the stirring pin further increased, many groove defects appeared again. This can be attributed to the close correlation between thermal input and the welding parameters. At low rotation speeds, both the thermal input and the metal softening degree were insufficient, which led to poor mobility and the appearance of a few groove defects on the surface of the front side. As the stirring pin’s rotation speed increased, the metal-softening region increased, and the groove defects disappeared gradually. With the further increase of the stirring pin’s rotation speed, a large proportion of the weld metal was inhaled into the gap between the stirring pin and the shaft shoulder along the axis direction. Therefore, no sufficient metal was filled on the front side, thereby leading to the appearance of groove defects on the weld surface. Therefore, well-formed welds could be obtained under reasonable technological parameters.

### 3.3. Effect of Rotation Speed on Macro- and Microstructures of SSFSW T-Joints

**Figure 8:**
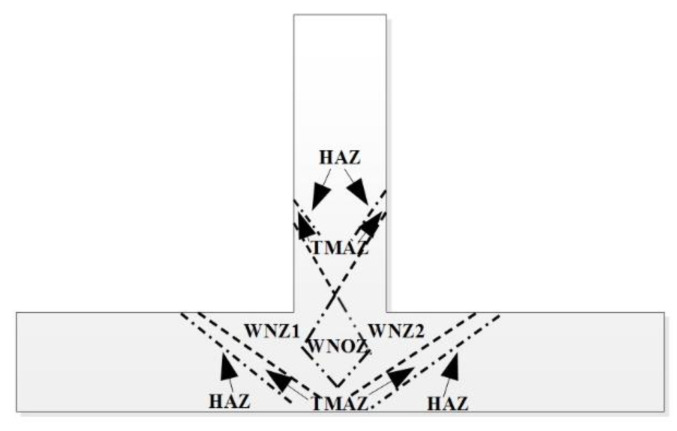
The different zones of the SSFSW T-joint.

**Figure 9:**
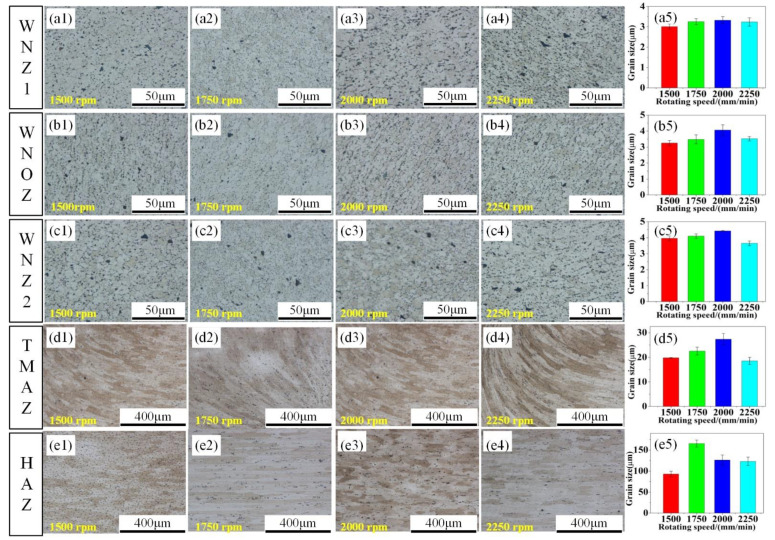
The microstructures and average sizes in the T-joints welded via SSFSW at different rotation speeds of the stirring pin: (**a1**)–(**e1**) 1500 rpm; (**a1**)–(**e2**) 1750 rpm; (**a1**)–(**e3**) 2000 rpm; (**a4**)–(**e4**) 2250 rpm;

The SSFSW T-joint sections include WNZ (the WNZ can be divided into the WNZ1, the WNOZ (Weld nugget overlap zone), and the WNZ2), TMAZ and HAZ, as can be seen in Figure 8. Figure 9 displays microstructures and average sizes in the T-joint welded via SSFSW at different rotation speeds, at a fixed welding speed of 50 mm/min. The materials in the WNZ were subjected to violent stirring action by the stirring pin, and the original structure underwent intense thermo-mechanical coupling action, thereby leading to recovery and recrystallization of WNZ grains. The WNZ was full of fine equiaxed grains. It should be noted that the average grain size was the largest in the WNZ2, followed by the WNOZ and the smallest grains were in the WNZ1. This may be due to the fact that the deformation of the first weld seam reduced the heat conduction efficiency of the stirring pin during the welding of the second seam, thereby failing to provide sufficient heat in time and significantly weakening the recrystallization degree of grains in this region. The TMAZ was subjected to shear action induced by the material plastic flow around the stirring pin and weld thermal cycles to a certain degree during the welding process. Accordingly, the grains were elongated and deformed along the direction of the maximum shear force. The HAZ was only subjected to weld thermal cycles without plastic deformation and dynamic recrystallization of grains. The grains in HAZ underwent coarsening to a certain degree under the thermal weld cycle.

The deformation state of grains in Al alloy is connected with the deformation rate and the deformation temperature. The final size of the grains in the SSFSW T-joint can be regarded as the result of competition and equilibrium between grain refinement and grain growth. The mean grain size of each region all first increased and then dropped with the increase of the rotation speed of the stirring pin in the SSFSW T-joints. This is because the change of the stirring pin’s rotation speed can simultaneously change both material’s deformation rate and temperature. As the rotation speed of the stirring pin changed increased from 1500 to 2000 rpm, the deformation temperature during the welding process rose rapidly. Under the stirring action of the stirring pin, the crystal grains can grow at high temperatures in a short time, and therefore, the mean grain size in the T-joints increased with the increase of the rotation speed. At a high rotation speed of 2000–2250 rpm, the rotation speed-induced temperature rise was not obvious. Simultaneously, the deformation rate of the material began to increase, accompanied by the decline in recrystallization trend and gradual decrease of the mean grain size.

### 3.4. Effect of Rotation Speed on the Mechanical Properties of T-Joints Welded by SSFSW

**Figure 10:**
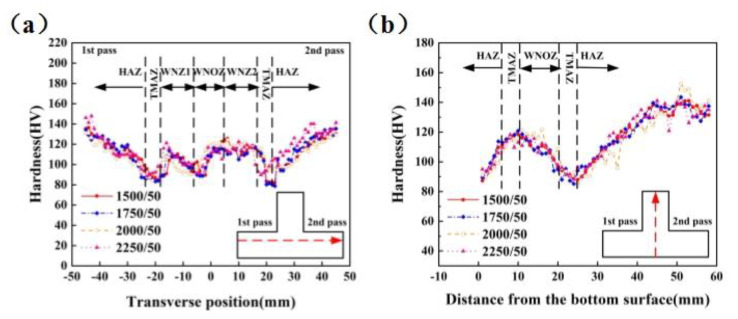
Hardness variation with rotation speed in different plates: (**a**) base plate and (**b**) rib plate.

**Figure 11:**
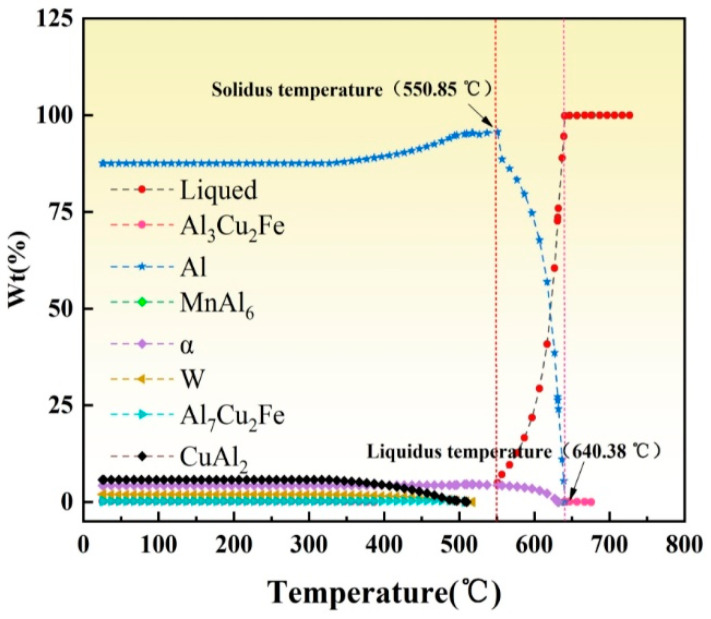
Evolution of precipitated phases in 2A14 Al-alloy with temperature.

**Figure 12:**
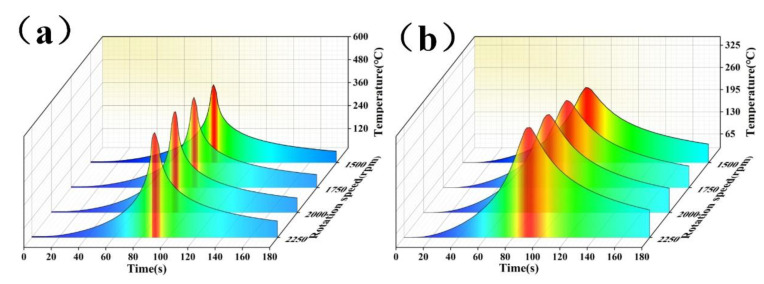
Temperature variation in various regions of welded T-joints at different rotation speeds: (**a**) WNZ, (**b**) TMAZ.

Figure 10 shows the hardness distribution patterns of the base and rib plates when the stirring pin’s rotation speed changed, while the welding speed was fixed at 50 mm/min. In particular, Figure 10a,b shows the hardness variation in the base and rib plates, respectively. The overall hardness distribution was W-shaped in the base plate. Hardness was the highest at both sides of the base material (BM) and dropped from the BM to the weld center, reaching its minimum in the TMAZ of the first welds and recovering in the WNZ. The overall hardness distribution in the rib plate was N-shaped, i.e., had an increase-decrease-increase trend. The measurement along the rib plate’s centerline only passed through the WNOZ, while the lowest hardness was observed in the TMAZ. As regards 2A14 Al alloy, the precipitation was the main strengthening mode. In particular, the α-solid solution, CuAl_2_ (*θ* phase), and Al_5_Cu_2_Mg_8_Si_6_ (*W* phase) played dominant roles. Figure 11 shows the precipitated phase variation with temperature. The peak temperature variation trends in the WNZ and TMAZ at different rotation speeds are shown in Figure 12. The peak temperatures in the WNZ increased from 427.13 to 568.10 °C, and the peak temperatures in the TMAZ were always lower than the values in the WNZ, as the peak temperature in the TMAZ increased linearly from 245.08 to 346.67 °C at a significantly lower temperature-rising rate than the value in the WNZ. According to the temperature field’s simulation results, peak temperatures in the HAZ and TMAZ were lower than that of the solid solution of the precipitated phase. Under thermal cycles, original grains were deformed, while the precipitated phase underwent growth and coarsening. Therefore, the HAZ hardness in the TMAZ vicinity dropped to its minimum. Under high-temperature thermal cycles, the original CuAl_2_-phase was fully solved, and the α-solid solution was partly solved. In the subsequent cooling process, atoms were reorganized to reform other types of precipitate phases. The WNZ1 hardness was slightly lower than that of the WNZ2, which can be attributed to the growth and coarsening of precipitated phases in the WNZ1 under the second welding process’s thermal input.

As also shown in Figure 10, the base and rib plates’ hardness distributions exhibited no obvious variation with rotation speed. When the latter varied within a range of 1500–1750 rpm, the base plate’s lowest hardness was observed in the TMAZ of the first weld. As the rotation speed exceeded 2000 rpm, the base plate’s lowest hardness appeared in the TMAZ of the second weld. The hardness of the rib plate showed an N-shaped distribution pattern. The lowest hardness was also located in the TMAZ. This is due to the fact that the thermal input of the first welding process could hardly sustain the formation of precipitated phases in the TMAZ of the first weld at a rotation speed of 1500–1750 rpm. The latter resulted in the coarsening of the original precipitated phases. As the rotation speed grew to 2000 rpm, an increase in the thermal input promoted the formation of precipitated phases in the TMAZ of the first weld, thereby leading to a slight increase in the TMAZ hardness of the first weld.

**Figure 13:**
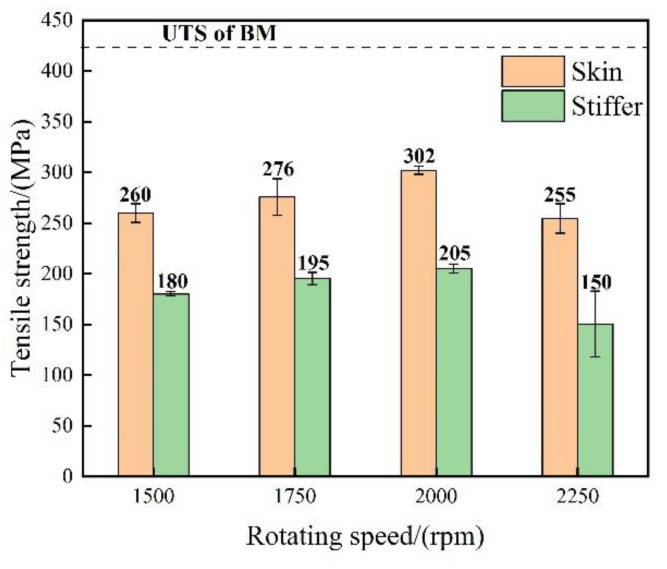
Tensile properties of the T-joint welded by SSFSW at different rotation speeds of the stirring pin.

Figure 13 shows the base, and rib plates’ tensile strengths of the SSFSW T-joints welded at a welding speed of 50 mm/min and different rotation speeds of the stirring pin. These tensile strength values were all lower than that of the BM (424 MPa). As the stirring pin’s rotation speed increased, the tensile strengths of the base and rib plates all first increased and then dropped. At a rotation speed of 2000 rpm, the base and rib plates’ tensile strengths reached their maximum values of 302 and 205 MPa, respectively, which were approximately 71.21% and 48.34% of the BM. Since the material plasticity was low at low rotation speeds, the material failed to fill the cavity at the back of the stirring pin during the welding process, and a series of surface defects appeared. The latter defects served as stress raisers during tensile tests and deteriorated the material’s mechanical properties. When the rotation speed was increased, the metal-softening region expanded, and the peak temperature in the weld also rose. However, a large peak temperature increased post-weld tensile residual stresses, which deteriorated the T-joint’s mechanical properties and contributed to the tensile strength decline.

**Figure 14:**
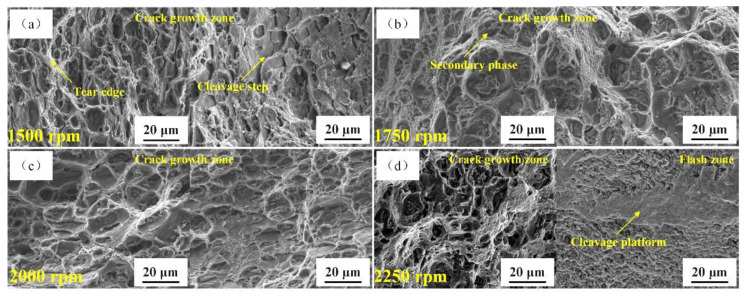
Fracture morphologies of the T-joint’s base plate at different rotation speeds of the stirring pin: (**a**) 1500 rpm; (**b**) 1750 rpm; (**c**) 2000 rpm; (**d**) 2250 rpm.

Figure 14 shows the fracture micromorphology of the base plate at different rotation speeds of the stirring pin and a 50 mm/min welding speed. At a rotation speed of the stirring pin of 1500 rpm, some shallow dimples and a few torn edges could be observed at the fracture surface, while some cleavage platforms existed on the cleavage platform. When the stirring pin’s rotation speed rose to 1750 rpm, the number of dimples increased, and a large number of the secondary-phase particles and high torn edges appeared in the dimples. The secondary-phase particles hindered the slippage, causing stress concentration at the boundary between the second-phase particles and the slip surface. As soon as stresses reached the peeling strength level, the secondary-phase particles were peeled from the matrix, thereby enhancing the plasticity. As the stirring pin’s rotation speed increased to 2000 rpm, the fracture was full of large dimples. The number of secondary-phase particles in the dimples increased significantly, which contributed to plastic deformation and further enhanced the plasticity. As the rotation speed of the stirring pin further increased, some cleavage platforms appeared on the fracture’s lower surface. The number of dimples dropped obviously, accompanied by the decline in plasticity. Overall, as the stirring pin’s rotation speed increased, the plasticity of the base plate first increased and then dropped, while the fracture mode gradually changed from ductile to ductile-brittle mixed-mode.

**Figure 15:**
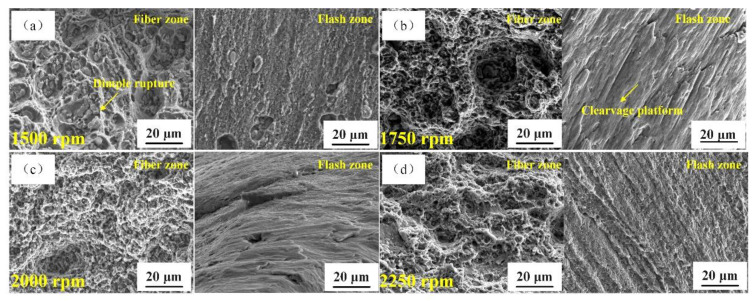
Fracture morphologies of the T-joint’s rib plate at different rotation speeds of the stirring pin: (**a**) 1500 rpm; (**b**) 1750 rpm; (**c**) 2000 rpm; (**d**) 2250 rpm.

Figure 15 depicts the rib plate’s fracture micromorphology at different rotation speeds of the stirring pin and a welding speed of 50 mm/min. At a rotation speed of the stirring pin of 1500 rpm, some small dimples with secondary-phase particles appeared at the fracture surface. As the stirring pin’s rotation speed increased to 1750 rpm, continuous dimples and obvious cleavage platforms simultaneously existed at the fracture surface. As the rotation speed increased to 2000 rpm, the number of dimples increased, while the number of cleavage platforms dropped, and the plasticity increased obviously. Finally, the number of dimples was reduced, and plasticity decreased with a further increase in the stirring pin’s rotation speed. Overall, the rib plate plasticity showed a similar variation trend with that of the base plate, and a typical ductile-brittle mixed-mode fracture was observed.

The results obtained above have shown that the stationary shoulder slid over the joint surface, and it had a cooling effect on the joint and acted as a heat sink; therefore, the “arc line” phenomenon and flashes could be avoided. Stirring pin rotation speed played an important role in the microstructure and mechanical properties of 2A14-T4 alloy SSFSW T-joints. At low rotation speeds, a small number of grooves appeared on the surface of the first and second welds. As the rotation speed increased to 2000 rpm, the first and second welds had smooth surfaces, and no surface defects appeared. However, as the rotation speed of the stirring pin further increased, many groove defects appeared. The grooves on the surface of the welds become a weak stress zone, reducing the T joint's mechanical properties.

It should be noted that twice the welding processes are needed in the joining of T-joint by SSFSW technology. In such a process, the weld nugget overlap zone should be required to avoid the creation of lack of fusion. As the study of microstructure and mechanical properties in the WNZ1, WNZ2, and WNOZ will reveal a more important value. The results obtained above had shown that the WNZ was full of fine equiaxed grains, the average grain size was the largest in the WNZ2, followed by the WNOZ, and the smallest grains were in theWNZ1. This may be because the deformation of the first weld seam reduced the heat conduction efficiency of the stirring pin during the welding of the second seam, thereby failing to provide sufficient heat in time and significantly weakening the recrystallization degree of grains in this region. The WNZ1 hardness was slightly lower than that of the WNZ2, which can be attributed to the growth and coarsening of precipitated phases in the WNZ1 under the second welding process’s thermal input. As also reported by Sun et al. [37], the residual stresses peak tensile regions are not symmetrically distributed, being the largest in the vertical stiffener near the position of the advancing side of the SSFSW pin in the second welds. This may be because the substantial stress relaxation could occur in the stress field introduced by the first welds due to the stress relief arising from the thermal field associated with the second welds.

## 4. Conclusions

(1) The arc lines and the flashes could hardly be observed on both welds’ surfaces in the SSFSW T-joints. At high rotation speeds of the stirring pin, multiple groove defects appeared on both welds’ surfaces, which became more serious with an increase in the rotation speed of the stirring pin.
(2) The WNZ was composed of isometric crystal grains. The average grain size of the WNZ2 was the largest, followed by that of the WNOZ, while that in the WNZ1 was the smallest. The TMAZ was composed of elongated grains, while the HAZ showed similar long-chunk rolled grains. As the stirring pin’s rotation speed increased, the mean grain sizes in the WNZ, TMAZ, and HAZ first increased and then decreased.
(3) The base and rib plates’ hardness distributions exhibited W- and N-shaped patterns, respectively. The coarsening of the secondary-phase particles during the welding process caused a decline in the WNZ hardness. The lowest hardness appeared at the boundary between the HAZ and TMAZ.
(4) With an increase in the rotation speed of the stirring pin, the tensile strengths of the base and rib plates first increased and then dropped, and the plasticity of the base and rib plates first increased and then decreased. Regarding the fracture mode, the ductile fracture dominated the base plate, and the brittle/ductile mixed fracture dominated the rib one.

## Figures and Tables

**Table 1:** Chemical composition range and mechanical properties of 2A14 aluminum alloy.

Material	Chemical Composition (wt%)									Mechanical Properties		
2A14-T4	Al	Cu	Mg	Si	Zn	Ti	Ni	Mn	Fe	Tensile strength (MPa)	Elongation (%)	Micro-hardness (HV)
	Bal	3.9–4.8	04–0.8	0.6–1.2	0.3	0.15	0.1	0.4–1.0	0.7	424	15–17	120–125

**Table 2:** Physical property parameters of 2A14-T4 Al-alloy.

*ρ*/(kg/m^3^) [25]	*T_m_*/(°C) [25]	*ε*/(°C^−1^) [25,26]	E/(GPa) [26]	ν
2840	640.38	2.44 × 10^−5^	73.8	0.33

**Table 3:** Thermophysical parameters of 2A14-T4 Al-alloy.

Temperature/°C	25 [26,27]	100 [26]	200 [26]	300 [26,27]	400 [27]	500	600
Thermal conductivity/[W/(m·°C)]	159	167	176	180	180	180	138.5
Capacity/[J/(kg·°C)]	837	837	879	963	1089	1120	3660

**Table 4:** Johnson–Cook constitutive equation parameters of 2A14-T4 Al-alloy.

Material Strength/MPa	Material Hardening Effect Constant/MPa [26,28,29]	Material Hardening Index [26,29]	Material Thermal Softening Coefficient [30,31]	Material Transition tempeature/°C [31]	Material mlting temperture/°C [25]
453	453	0.5948	1.08	25	640.38

**Table 5:** Welding parameters of the SSFSW of 2A14 Al-alloy T-joints.

Specimen No.	Welding Speed (mm/min)	Rotation Speeds (rpm)	Pin Tool Plunge Depth (mm)
1	50	1500	4.59
2		1750	
3		2000	
4		2250	

## Data Availability

The data presented in this study are available on request from the corresponding author.

## Abbreviations

FSW	Friction stir welding
SSFSW	Stationary shoulder friction stir welding
1st welds	First welds
2nd welds	Second welds
WNZ	Weld nugget zone
WNZ1	First weld nugget zone
WNZ2	Second weld nugget zone
WNOZ	Weld nugget overlap zone
HAZ	Heat affect zone
TMAZ	Thermo-mechanically affected zone
